# Supplementary material for: Squalene-Based Nano-Assemblies Improve the Pro-Autophagic Activity of Trehalose
Source: Pharmaceutics. 2022 Apr 14;14(4):862. doi: 10.3390/pharmaceutics14040862 (PMC9032118; doi:10.3390/pharmaceutics14040862)

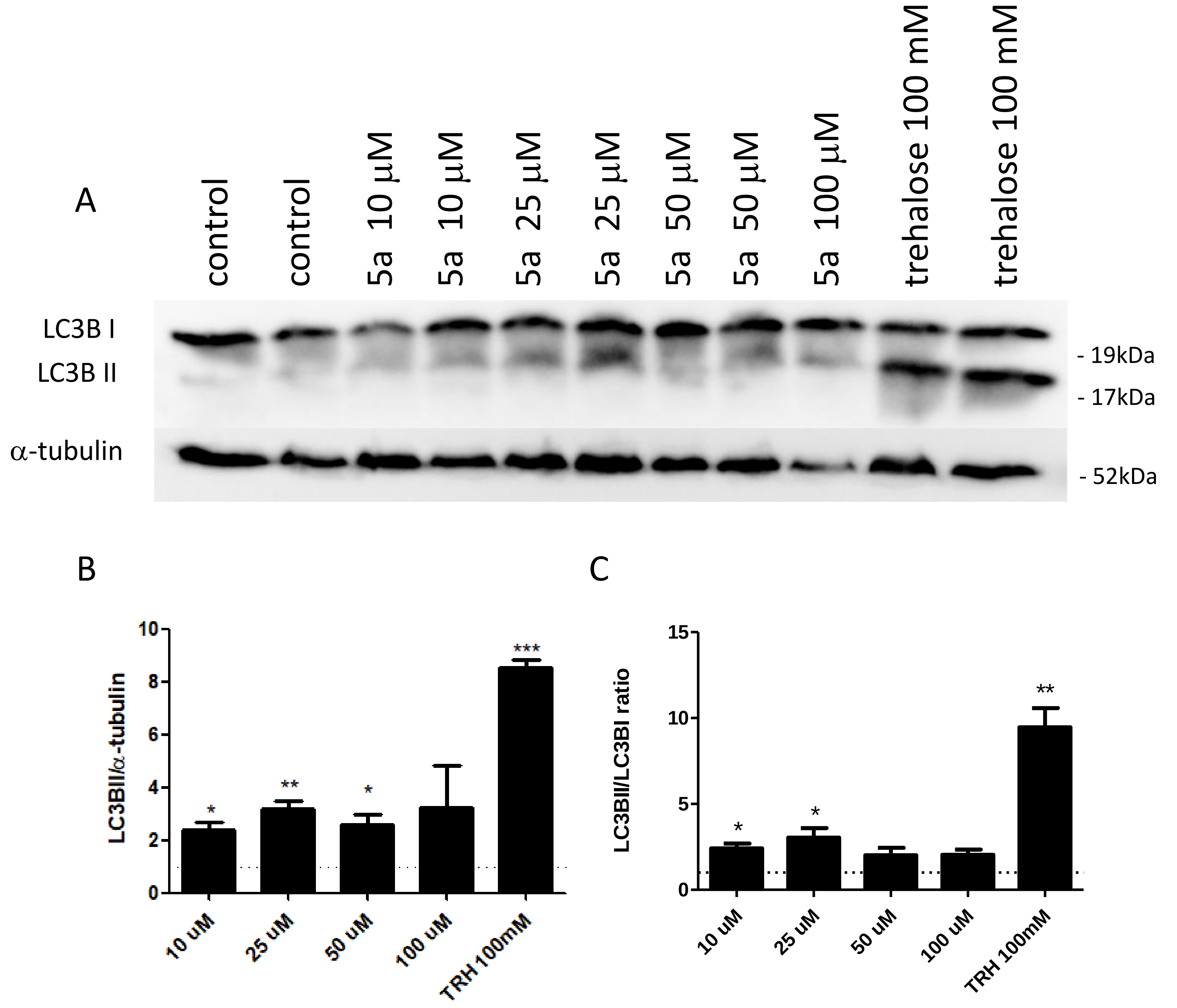

(A) Evaluation of autophagy induction in HEK293 cells treated for 24 hours with trehalose and **5a**. Upon treatment, we analyzed tubulin, LC3BI and II levels by western blotting.

(B) The graph shows LC3BII amount expressed as fold over α-tubulin. Data are expressed as mean ± S.E.; *< p<0.05, ** p<0.01, *** p<0.001 versus control, n=4-5.

(C) The graph shows LC3BII/LC3BI ratio. Each value was normalized on proper control. Data are expressed as mean ± S.E.; *< p<0.05, ** p<0.01 versus control, n=4-5.

Supplementary figure S1

Figure S2A

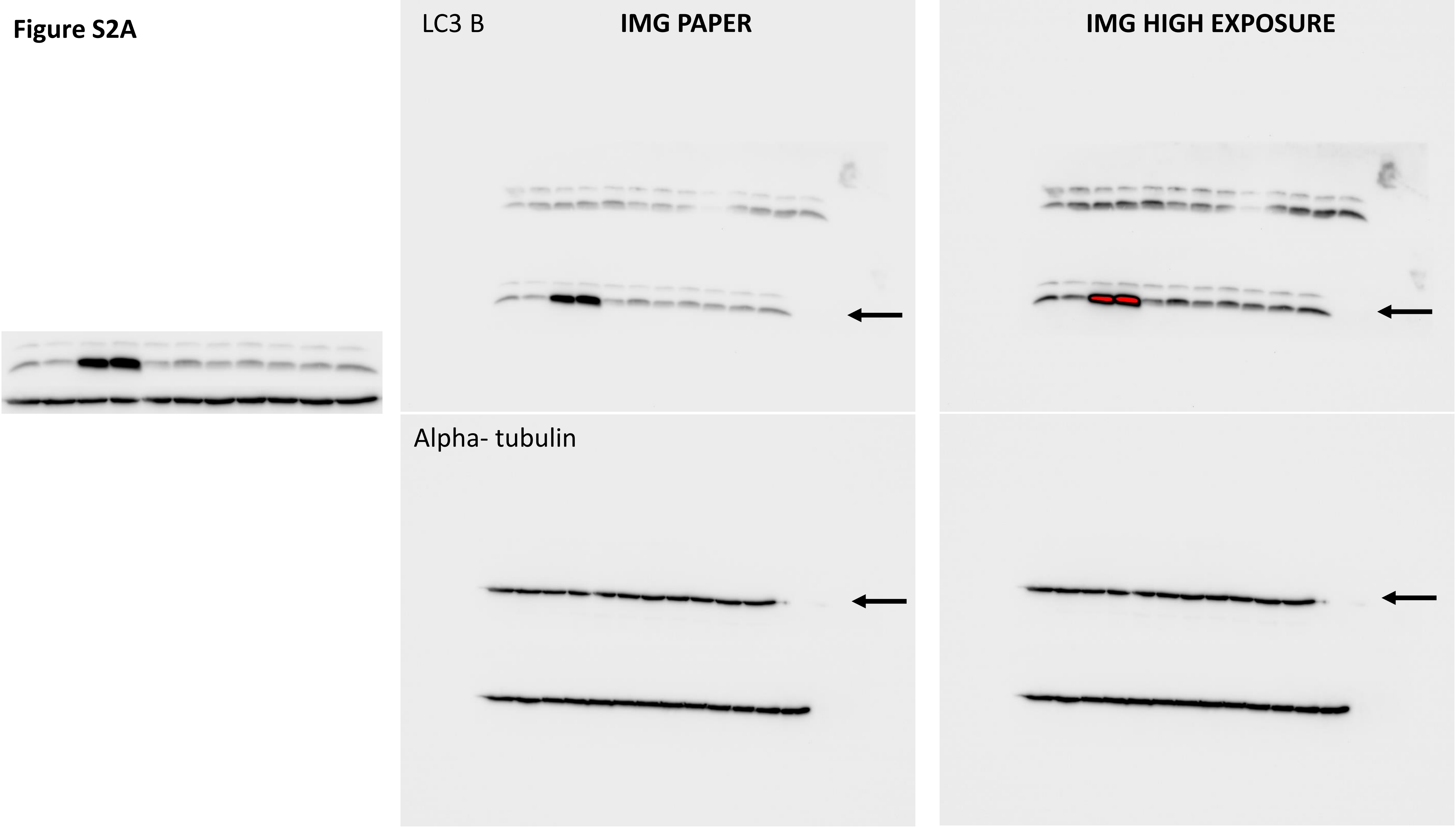

Figure S2B

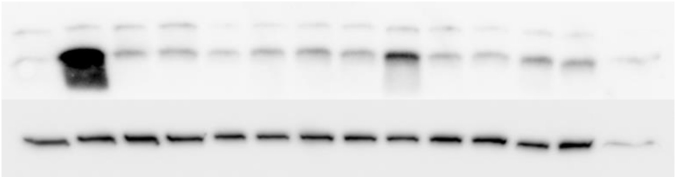

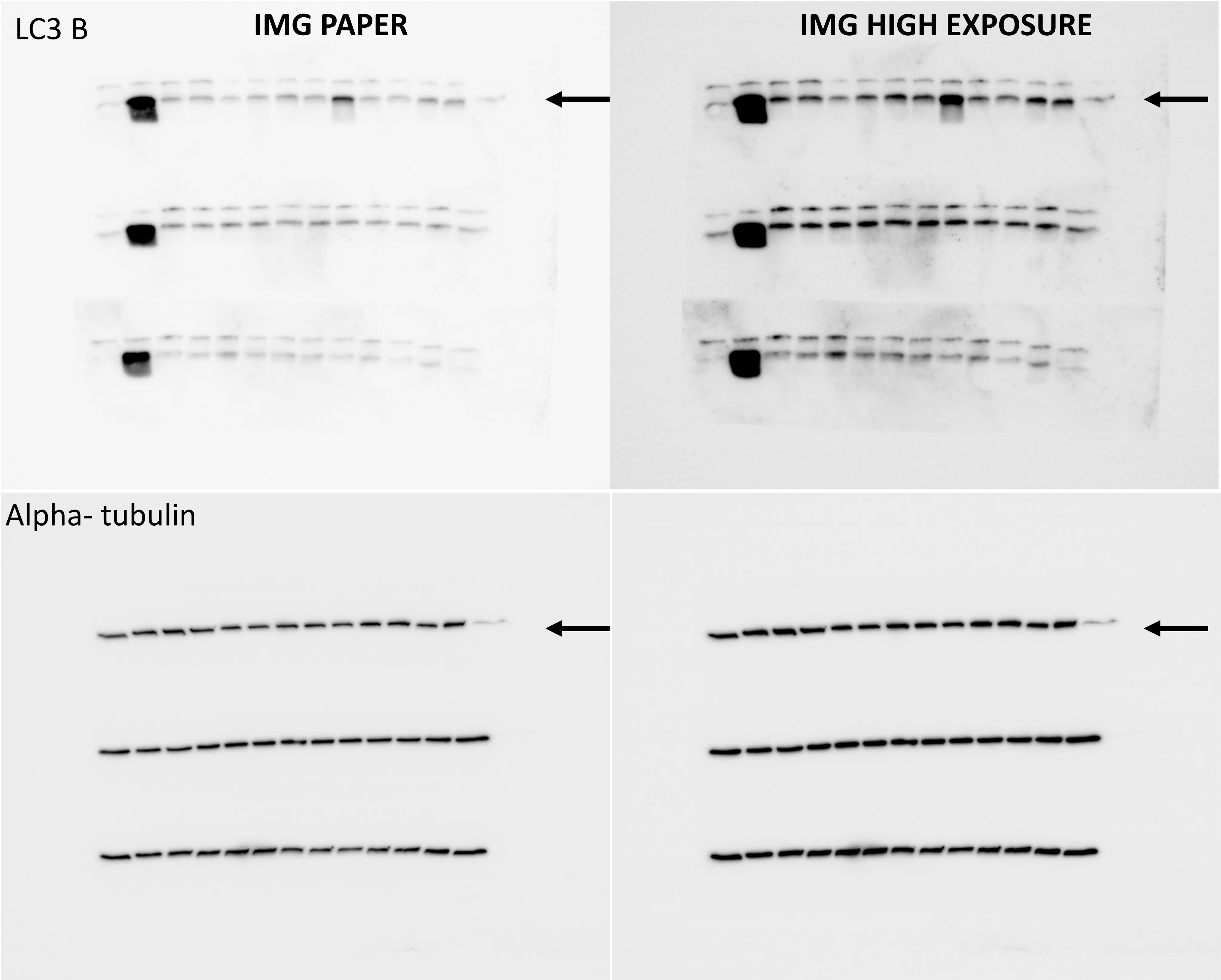

Figure S2C

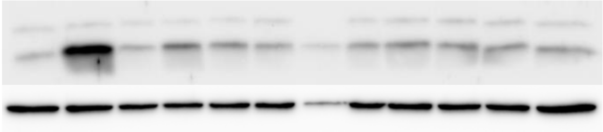

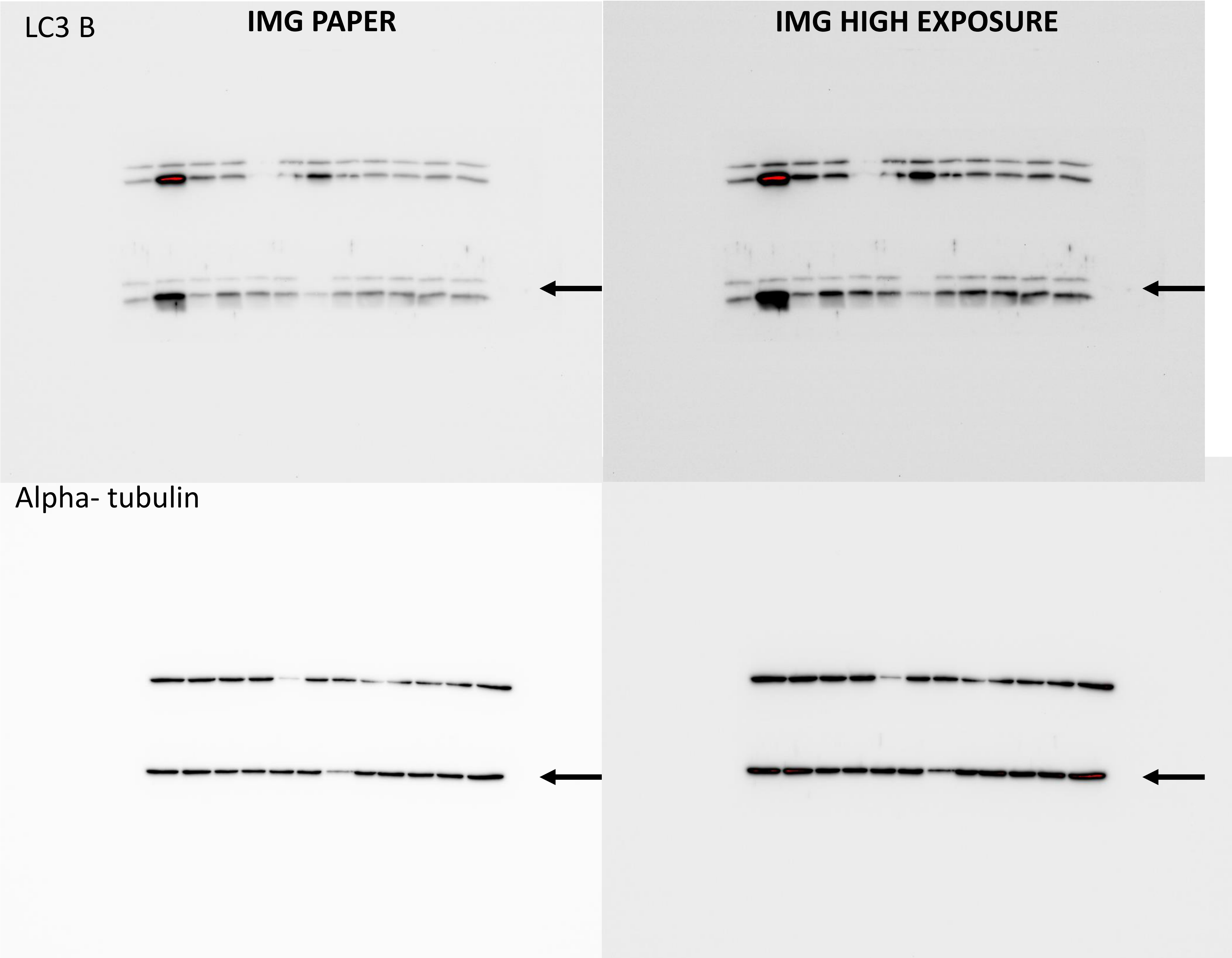

Figure S3

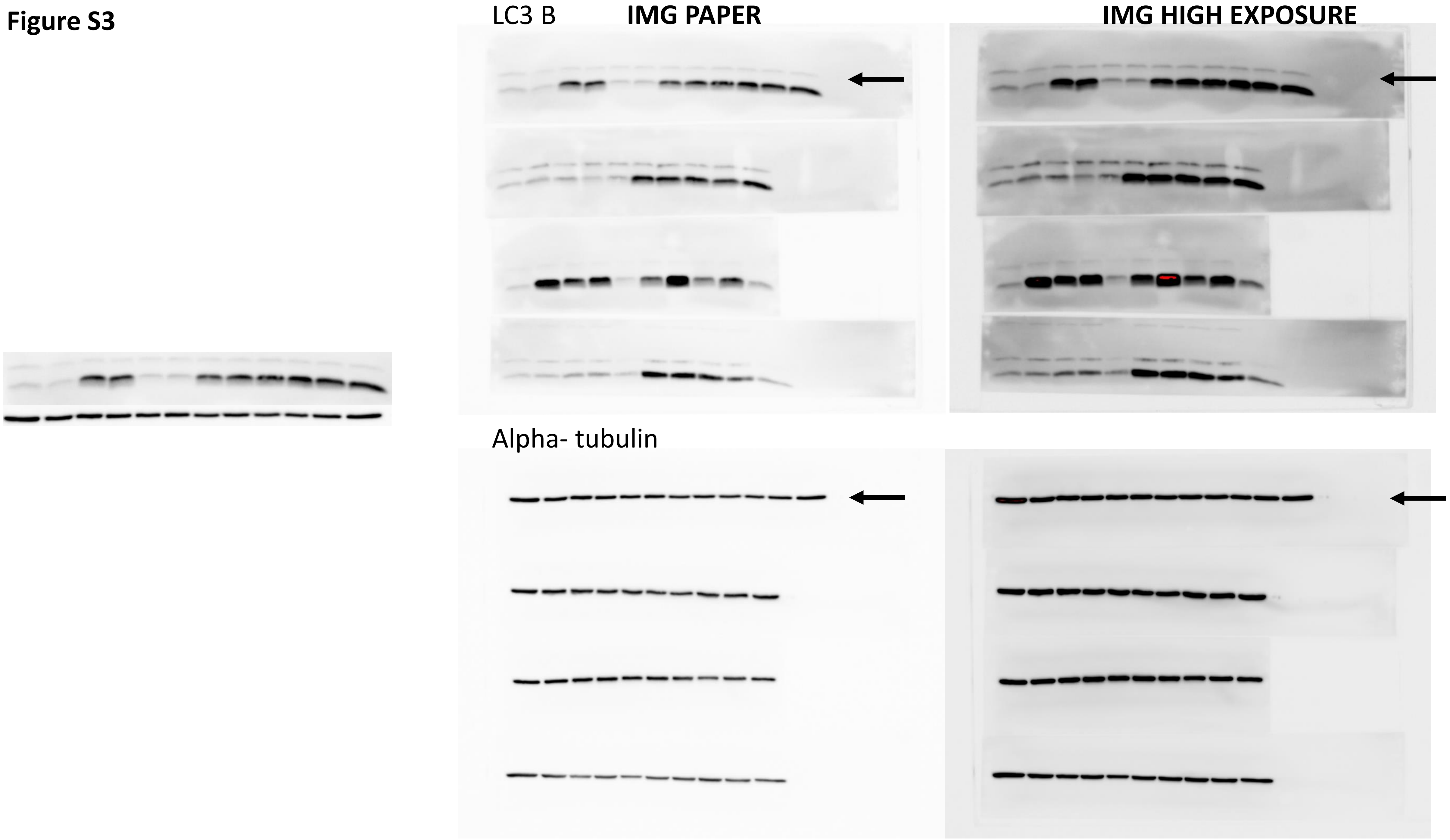

Figure S4

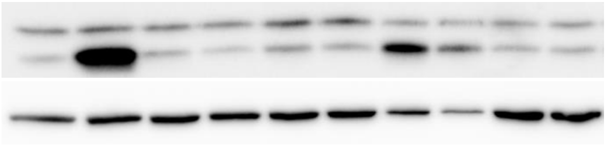

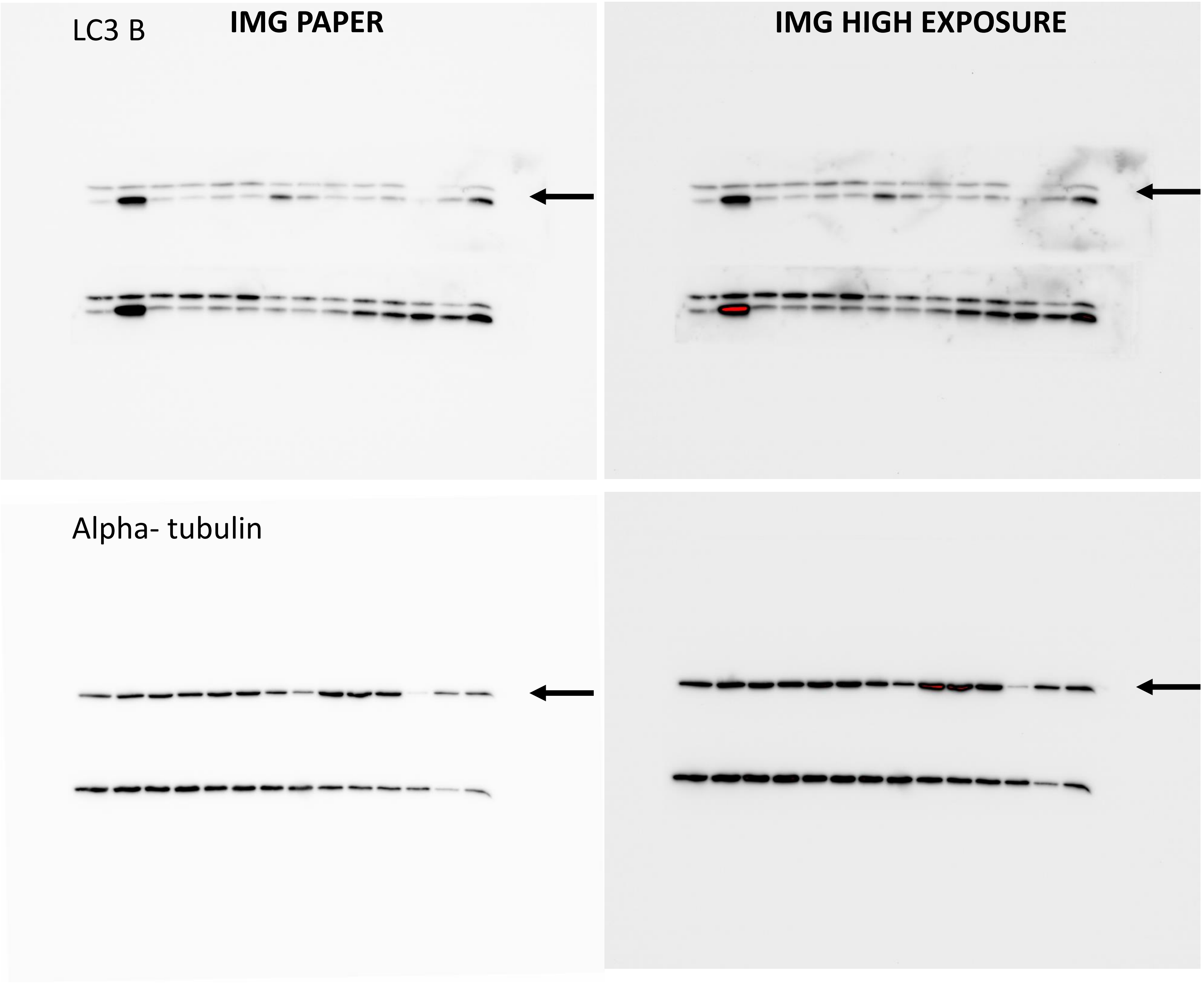

Supplement: Supplementary file 1 [file pharmaceutics-14-00862-s001.zip › pharmaceutics-1494321-supplementary.pdf]
